# The first regional interdisciplinary paediatric palliative care (PPC) workshop for the Eastern Mediterranean Region: a groundbreaking collective step for PPC integration

**DOI:** 10.3332/ecancer.2025.1948

**Published:** 2025-07-17

**Authors:** Rima Saad Rassam, Ryan R Lion, Siham Cherkaoui, Laila Hessissen, Ximena Garcia-Quintero, Lama Sayegh Najjar, Dolly Noun, Janane Hanna, Rana Yamout, Shahzadi Resham, Khaled Al Habaiba, Anwar Al-Nassan, Joe El-Khoury, Spandana Rayala, Qutaibah Alotaibi, Nahla Gafer, Giuseppe Troisi, Julia Downing, Suheir Rasul, Sima Jeha, Monnie Abraham, Michael J McNeil

**Affiliations:** 1 Department of Global Pediatric Medicine, St. Jude Children’s Research Hospital, Memphis, TN 38105, USA; 2 Hematology and Pediatric Oncology Department, August 20 Hospital, Ibn Rochd University Hospital, Casablanca 20000, Morocco; 3 Societé Marocaine D’Hématologie et d’Oncologie Pédiatrique, Rabat 10000, Morocco; 4 Pediatric Haematology and Oncology Center, Rabat Children's Hospital, Mohammed V University, Rabat 10000, Morocco; 5 American Lebanese Syrian Associated Charities, Memphis, TN 38105, USA; 6 Department of Pediatrics, Children's Cancer Institute, American University of Beirut Medical Center, Beirut 1107 2020, Lebanon; 7 Department of Nursing, American University of Beirut Medical Center, Beirut 1107 2020, Lebanon; 8 Balsam—The Lebanese Center for Palliative Care, Beirut 1107 2020, Lebanon; 9 Department of Internal Medicine, Palliative and Supportive Care Program, NKBCI, AUBMC, Beirut 1107 2020, Lebanon; 10 Department of Oncology, Aga Khan University, Karachi 74800, Pakistan; 11 Department of Pediatric and Child Health, Aga Khan University, Karachi 74800, Pakistan; 12 Pediatric Hematology-Oncology Unit, Tawam Hospital, Abu Dhabi 15258, United Arab Emirates; 13 Department of Pediatrics, King Hussein Cancer Center, Amman 11941, Jordan; 14 Palliative Care Department, Centre Hospitalier Public d’Hauteville, Plateau D’Hauteville 01110, France; 15 Sunflower Children’s Network, Two Worlds Cancer Collaboration, Vancouver V6T 1Z4, Canada; 16 Department of Pediatrics, Al Adan Hospital, Kuwait Ministry of Health, Hadiya 60000, Kuwait; 17 World Health Organization Eastern Mediterranean Regional Office, Cairo 11711, Egypt; 18 International Children’s Palliative Care Network, Suite 1b, Whitefrairs, Lewins Mead, Bristol BS1 2NT, UK; a https://orcid.org/0000-0002-8232-8733; b https://orcid.org/0000-0003-4524-1935; c https://orcid.org/0000-0003-4115-0374; d https://orcid.org/0000-0002-3322-273X; e https://orcid.org/0000-0003-1416-544X; f https://orcid.org/0009-0000-6260-8853; g https://orcid.org/0009-0000-4191-9513; h https://orcid.org/0009-0005-4970-242X; i https://orcid.org/0000-0002-9739-2164; j https://orcid.org/0009-0001-3969-7381; k https://orcid.org/0009-0002-0413-026X; l https://orcid.org/0000-0002-2246-4100; m https://orcid.org/0000-0003-1101-1074; n https://orcid.org/0000-0002-2148-4732; o https://orcid.org/0000-0002-0240-2076; p https://orcid.org/0000-0001-6291-093X; q https://orcid.org/0000-0002-2264-8604; r https://orcid.org/0000-0002-3450-785X; s https://orcid.org/0009-0001-3914-2394; t https://orcid.org/0000-0003-2835-5869; u https://orcid.org/0000-0003-1053-2812; v https://orcid.org/0000-0001-8817-1995

**Keywords:** palliative care, cancer care, paediatrics, oncology, hospice and palliative care nursing, quality of life, developing countries, capacity building, pain, Middle East, Eastern Mediterranean, interdisciplinary health team

## Abstract

Amidst the global disparities in providing Paediatric Palliative Care (PPC), the compounded realities in the Eastern Mediterranean region intensify the need for palliative care for children with cancer. This region hosts 12% of children needing PPC worldwide within limited specialised services, training and resources exacerbated by political instabilities. Immediate effective responses may reside in promoting interdisciplinary capacity-building combined with action planning among healthcare professionals, stakeholders and advocates for patients and their families. In response to these pressing needs, the First Regional Interdisciplinary PPC Workshop was held in-person in Rabat, Morocco on 6–8 November 2024, aiming to strengthen regional interdisciplinary healthcare professionals’ capacity through training and collaborative action planning to improve PPC integration in cancer treatment. Eighty-five attendees representing 15 countries including regional healthcare professionals, international experts, foundation representatives and policymakers united in the commitment to promote PPC integration and enhance the quality of life for children with palliative care needs, particularly those with cancer living in this region. Over 3 days, participants engaged in training, discussions and action planning. Day one provided essential skills to deliver PPC at the patient care level (micro-level), day two discussed institutional implementation of PPC services (meso-level), day three tackled national and regional endeavours to promote PPC integration (macro-level). At the conclusion of the workshop, participants gained knowledge and confidence in various PPC skills. They shared their plans to establish PPC teams/units, apply symptom management and communication skills, train colleagues within their settings, conduct research and maintain networking to encourage collaboration opportunities. The workshop marked a pivotal step to deepen understanding of the PPC landscape and resources in the region. This impactful experience laid the foundations for different opportunities in practice, research, policy and advocacy to accelerate PPC integration in the Eastern Mediterranean region, fostering collective efforts to improve childhood cancer care globally.

## Introduction

For over two decades, paediatric palliative care (PPC) has been an evolving field for children diagnosed with cancer and other serious illnesses [1]. Focused on enhancing quality of life, this comprehensive and compassionate care aims to alleviate the physical, psychological, social and spiritual suffering of children with serious illnesses and their families using an interdisciplinary approach [2]. In 2014, the World Health Assembly (WHA) declared palliative care as an ‘ethical responsibility of health systems’ [3]. However, access to palliative care remains a major challenge for people living in low- and middle-income countries, especially children [4]. The 2018 Lancet commission reported that yearly around 2.5 million children die with serious health-related suffering and more than 98% of these deaths occur in LMICs [5]. The majority of these children lack access to quality PPC, emphasising the need for the development of PPC in these regions [5]. Within these global disparities, the Eastern Mediterranean region hosts 12% of children in need of palliative care worldwide [6]. To address this limited progress of the WHA resolution, the recent World Innovation Summit for Health in collaboration with the World Health Organisation (WHO) recommended leveraging collaborations and ‘targeted interventions’ to accelerate the essential components for palliative care development such as national policies, community engagement, access to care and medicine, research and education [7] which reflect the components of the WHO conceptual model for palliative care development [8].

PPC is insufficiently developed in the Eastern Mediterranean region and, when present, is limited to a handful of capacity-building activities and isolated provision of services [9]. This region’s unique cultural, religious, socio-economic and geopolitical contexts present distinct challenges in delivering palliative care for children with serious illness. Many countries lack sufficient infrastructure, resources, supportive legislations, access to pain medication and trained healthcare professionals to provide comprehensive PPC [10–12]. The barriers vary across the region. For example, in Iraq, the absence of national policies supporting PPC such as opioids use and do-not-resuscitate orders results in limited access to services for children in need. In Egypt, the availability of structured PPC training for healthcare professionals is limited. The displacement due to ongoing conflicts in Syria and Palestine stretches the healthcare systems in countries with more resources like Jordan and Lebanon which intensifies the challenges faced by children with cancer and their families in affected countries [13, 14]. These realities result in exacerbated suffering in children and call for immediate and multifaceted actions to timely mobilise efforts at all levels.

One of the effective responses tailored to the regional needs resides in promoting interdisciplinary capacity-building initiatives [15]. The development of high-quality palliative care, in part, relies on adequate training of all healthcare professionals involved in the care of patients [8]. In addition, the WHO through the Global Initiative for Childhood Cancer (GICC) [16], have supported previous PPC efforts in the Eastern Mediterranean Region by providing guidance and technical support, and facilitating regional collaboration to develop sustainable palliative care programmes. Within cancer care, foundations and non-governmental organisations play a vital role in raising awareness, funding and advocating for policy change. When combined with a collaborative plan involving all stakeholders, training endeavours can accelerate and standardise PPC integration at the levels of the patient, the institution and the country/region.

In response to this pressing need for PPC integration in the Eastern Mediterranean region, the First Regional Interdisciplinary PPC Workshop was held in Rabat, Morocco on 6–8 November 2024, in alignment with the WHO Cure*All* Framework [17] for action to strengthen capacity in childhood cancer care, particularly with regards to palliative care. Unlike similar initiatives, this workshop distinguished itself by fostering a cross-border multi-level approach, uniting efforts of interdisciplinary clinicians, national and regional policymakers and non-governmental charitable organisations. This approach harmonised diverse perspectives on key PPC change drivers, sparking sustainable dialogue and collaborations for improving PPC integration in patient care, service implementation and national/regional advocacy. This report outlines the workshop’s design, execution and implications for the future of PPC development in the region.

## Design of the workshop

### Design, sample and setting

The workshop combined a comprehensive interdisciplinary PPC training and collaborative action planning for PPC development in the region. It was organised by St. Jude Global [18] (Palliative Care, Nursing and Eastern Mediterranean Region Programs) in collaboration with American Lebanese Syrian Associated Charities (ALSAC), the Pediatric Oncology East and Mediterranean (POEM) group [19, 20] and the Moroccan Society of Pediatric Hematology Oncology (SMHOP) [21]. The geographic focus of this workshop specifically targeted POEM member countries [19]. The scientific and logistic preparations of the workshop took place through periodic online meetings commencing in March 2024, to co-design the content and the participants’ selection process and to collectively plan and execute the activities. The selection criteria included: a minimum of 3 years of experience in paediatric haematology/oncology/palliative care, a strong interest in PPC, current involvement in leadership, training, quality improvement or research activities at your local institution and a willingness to share their experience with colleagues after attending the workshop.

The purpose of this initiative was to strengthen the capacity of interdisciplinary healthcare professionals and foundation representatives to enhance PPC integration in the region. The workshop engaged 85 attendees from various healthcare professions (physicians, nurses, psychologists, social workers, pharmacists), national governmental representatives and national/regional nongovernmental organisations and advocacy groups. Attendees included 52 participants from 12 regional countries who were selected through an application process (Table 1), along with 28 regional and international organisers and speakers from three additional countries (United States, United Kingdom and France) and five government representatives from Morocco. They represented St. Jude Global, ALSAC, POEM, SMHOP, WHO (Eastern Mediterranean Regional Office and Morocco Office), the International Children’s Palliative Care Network (ICPCN) [22] and the Franco-African Pediatric Oncology Group (GFAOP) [23]. Most participants were physicians (*n* = 23, 44%) followed by nurses (*n* = 19, 37%), allied health professionals (*n* = 7, 14%) and charitable foundations representatives (*n* = 3, 6%). Participants varied in experience, with nearly half having more than 5 years of experience in their roles (*n* = 25), highlighting the mixture of both seasoned and early-career professionals. Geographically, the highest representation was from Morocco (*n* = 19, 37%) as the host country, followed by Pakistan (*n* = 8, 15%). These demographic characteristics underscore the diverse mix of backgrounds that facilitated a rich exchange of experiences on PPC integration in the Eastern Mediterranean region. Despite the extraordinary challenges in the region, the organisers remained steadfast in continuing with the activity guided by the belief that PPC is needed now more than ever. However, the ongoing conflicts prevented some participants from attending the workshop.

**Figure 1.**
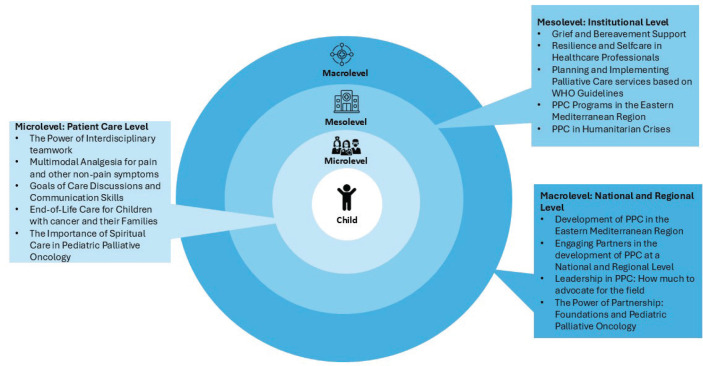
Workshop design using the ecological systems theory. Figure 1 depicts the workshop design and content of the 3 days based on three levels of the Ecological Systems Theory [24]. The ‘micro-level’ included patient care level topics, the ‘meso-level’ addressed the institutional processes and PPC services and the ‘macro-level’ discussed the national and regional policies and programmes related to PPC.

The workshop content was designed using the Ecological Systems Theory as a framework (Figure 1) [24]. The ‘micro-level’ encompassed the patient care level where healthcare providers interact with the child. The learning objectives in this level were to demonstrate an increased knowledge about PPC principles and develop practice skills in communication, symptom management and holistic care to immediately apply to clinical practice. The ‘meso-level’ entailed exploring the institutional processes enabling the provision of PPC services. The objective was to develop an initial plan for implementing improvement initiatives in PPC at the participants’ local institutions. The ‘macro-level’ pertains to the national and regional policies and programmes related to PPC. The objectives at this level were to identify priorities for advancing PPC in the region and promote interdisciplinary collaboration by collectively developing a regional action plan. As such, each day addressed one system level with its specific learning objectives to facilitate participants’ understanding of the dynamic interconnectedness of systems in PPC development and integration.

### Delivery methods

The workshop was held in-person with content facilitated by regional and international experts in PPC using interactive presentation methods and tools, such as role play and group activities. The spoken languages among attendees included English, French and Arabic. Although the main communication and presentations were in English, professional simultaneous translation services and trilingual presenters were available to ensure a meaningful engagement of all attendees.

### Workshop evaluation survey

At the conclusion of the workshop, the participants’ feedback was collected through an evaluation survey designed specifically for the workshop. The survey was self-administered electronically at the end of the workshop, in English and French. The survey combined satisfaction questions and evaluation of PPC learning. We sought participants’ input on the workshop content, delivery methods, travel logistics and areas for future improvement. In addition, we solicited participants’ insights on the extent to which the workshop fostered their knowledge and confidence in PPC skills and on their initial thoughts for plans to integrate PPC in their settings. The items’ format included multiple choice questions, 5-point Likert scales and open-ended questions. Thus, the survey helped examine the participants’ satisfaction with the workshop experience and the PPC growth gained from this experience.

## Summary of the workshop

### Opening of the workshop

The workshop began with introductions from the representatives of global, regional and local collaborators, highlighting how this workshop aligns with their bigger strategy. The representations encompassed senior leaders from the organising entities (St. Jude Children’s Research Hospital, POEM, SMHOP) and from GFAOP and WHO.

Mr Toby Kasper and Ms Suheir Rasul presented a high-level overview of St. Jude Global strategy to foster worldwide alliances and initiatives so that ‘every child with cancer, or blood disorder has access to quality care.’ The interdisciplinary nature of the workshop was reflected by the collaboration of three programmes within the St. Jude Global structure: the Eastern Mediterranean Region, Palliative Care and Nursing programmes. The directors of these programmes, Dr Sima Jeha, Dr Michael McNeil and Dr Monnie Abraham, briefly presented their respective initiatives.

The nursing chair of POEM group, Mr Khaled Al-Habaiba, highlighted their regional efforts to continuously ‘improve paediatric cancer care through capacity building training, research and advocacy.’ Then, the president of SMHOP, Professor Siham Cherkaoui, described the workshop as an opportunity to leverage the existing structure in Morocco. Professor Leila Hessissen highlighted the implication of this initiative beyond the Eastern Mediterranean region, extending to countries within GFAOP. The welcome remarks concluded with Dr Giuseppe Troisi and Dr Imane El Menchawy emphasising the WHO’s role in the implementation of the GICC in the Eastern Mediterranean Region and Morocco.

To facilitate the introduction of the attendees, the ALSAC team conducted an interactive activity on ‘elevator pitch’ [25]. Participants developed a 30-second pitch and practiced it amongst each other as a powerful means to showcase themselves and their organisations. The upfront engagement of all stakeholders created momentum amongst attendees and instilled a sense of commitment to optimise the workshop’s purpose.

### Day 1 – PPC at the micro-level

The scientific content of the first day aimed at enhancing participants’ knowledge and clinical skills for incorporating PPC principles in their daily practice with children and their families. Two introductory sessions set the stage for the 3 days. The overview of PPC in paediatric oncology, highlighted the specific PPC needs of children with cancer, the benefits of early integration of PPC in the care trajectory in parallel to curative treatment [26] and the strategies to shift the paradigm for ensuring access to high-quality PPC. The session on the importance of interdisciplinary teamwork, distinguished the unique aspects of this approach within PPC. Building on a myriad of expertise from medical and non-medical backgrounds, the interdisciplinary approach nurtures a collaborative environment and enhances powerful implications on patient care.

The management of symptoms was divided into three sessions addressing multimodal analgesia, non-pain physical symptoms and psychosocial symptoms. Three experts in the field reinforced the steps of symptom management. Through real-life case studies, participants explored the factors contributing to suffering, the importance of thorough systematic assessment and the art of using multiple modalities with pharmacological and non-pharmacological interventions. Particular attention was drawn to cultural implications for proper symptom management such as the stigma of opioid use and the influence of family dynamics.

The afternoon continued with the goals of care discussions and communication of serious news as fundamental PPC skills to foster hope. Participants learned the concept of ‘regoaling’ [27] and created a headliner sentence in their own language to help patients and families map out new goals when patient’s condition changes. They also engaged in a role play featuring high-quality communication to disclose difficult news [28]. This activity allowed participants to practice compassionate conversation skills in a culturally adapted manner.

The first day included a session on specific PPC needs at the end-of-life to address the changes in clinical status and the comfort measures during the last days of the child’s life. Participants shared their experiences with legal considerations such as the absence of laws or laws enforcing aggressive measures in some countries. The session was an eye opener to change the narrative of ‘*nothing can be done*’ to ‘*a lot can be done to comfort the child and family and ensure a dignified death*’.

The day ended with an insightful panel session on the importance of spirituality in PPC. The discussion clarified the overarching nature of spirituality that encompasses both existential and religious aspects intersecting with the cultural context. The spiritual diversity in the Eastern Mediterranean region calls for an individualised patient-centered approach to *‘meet the patients where they are’.*

The first day of the workshop provided a solid common understanding of PPC principles for clinical practice which served as a crucial foundation for subsequent workshop days. Below are key points summarising the PPC aspects discussed at the micro-level:

- Pediatric Palliative Care is most effective when integrated early in the disease trajectory. There is a regional need to correct misconceptions and raising awareness among healthcare professionals and non-healthcare professionals.
- An interdisciplinary approach is necessary in the clinical care to respond holistically and effectively to the patients’ and families’ needs.
- Culturally sensitive care and acknowledgement of distinct spiritual aspects in the region are pillars in addressing patients’ symptoms and navigating effective communication throughout the disease journey and at the end of life.

### Day 2 – PPC at the meso-level

The second day was focused on the ‘meso-level’ aspects of PPC pertaining to institutional processes, with particular emphasis on PPC service-level implementation. Through interactive didactic sessions and group activity, participants deepened their understanding of PPC principles with practical strategies to implement and/or improve PPC programmes.

The first session addressed the topic of grief and bereavement. This provided key insights into how healthcare workers can effectively support families both before and after a child’s death through an interdisciplinary lens [29, 30]. The discussions with the audience underscored the importance of early involvement in palliative care to enhance therapeutic relationships with bereaved families. The session also emphasised the need for institutional policies tailored to the cultural context to support effective implementation. The next session explored the topic of resilience and self-care strategies while acknowledging the emotionally demanding PPC roles and the regional instability. The discussion highlighted the benefits of compassion on patients/families, providers and the quality of care [31].

The remainder of the day covered the essential components for institutional implementation of PPC. A didactic session outlined the guidelines for the planning and implementation of palliative care services, bringing attention to various resources developed by the WHO that offer practical guidance for high-quality PPC services [8, 17, 32–42]. This was followed by a panel session showcasing successful PPC programmes from Jordan, Pakistan, Lebanon and Morocco. Comparing different strategies, each country tailored its PPC successful approach to its unique context and challenges. Jordan focused on leveraging PPC training to enhance healthcare professionals’ skills, which was a practical solution given the existing country's healthcare structure. As for Pakistan, building PPC teams was a local priority to respond to high demand within the limited available resources. For Lebanon, a viable solution facing the socio-economic challenges was to sustain home care through technology and teamwork, thus, ensuring continuous care for children despite limited hospital capacity. Morocco, on the other hand, mobilised policymakers and drew on experiences from adult palliative care to drive PPC integration into national healthcare reforms. Each strategy reflected the country’s PPC realities and, emphasised possible solutions from various angles (training, resource efficiency, technology and policy change). Key lessons underscored the importance of patience, realistic expectations and perseverance as reflected by Dr Cherkaoui stating, ‘*on s’est imposé pour ne pas laisser les enfants souffrir* (We stood strong to prevent children’s suffering).’ The panel’s practical insight reinforced that PPC in LMICs is possible and transformative when approached through innovation and determination.

The afternoon session put theory into practice with a collaborative activity where participants developed block diagrams outlining the tangible steps to establish or strengthen PPC services. The selected services included establishing PPC programmes, referral criteria, bereavement care, sustainability and home-based programmes. The groups’ presentations reiterated that standardisations of processes within institutional structures along with real-time impact analysis are attributes to success.

The day concluded with another panel session on delivering PPC in humanitarian crises. The regional panellists described the unique PPC challenges in conflict-affected areas within significant resource constraints. The exchange of experiences revealed ‘*realities of care that we don’t hear on the news and on television*.’ – Dr Dolly Noun (Lebanon). The panellists described the delicate balance of healthcare professionals’ own safety and quality patient care. This requires innovative, emergency-driven solutions including sourcing opioids, leveraging telehealth and optimising a compassionate approach among patients, families and healthcare professionals.

Overall, the second day consolidated the thoughtful discussion of PPC principles from an institutional angle. The interactive sessions provided a venue for discussing actionable measures to improve PPC services in various contexts. The three key takeaways from the day were:

- Compassion through PPC fosters meaningful therapeutic relationships with patients and families during difficult times and strengthens resilience among teams.
- Successful implementation of PPC services relies on context-specific strategies tailored to the patient/family needs, institutional culture and available resources.
- Harnessing innovation and resilience enhance the effectiveness and sustainability of PPC delivery and ensures high-quality care in both stable periods and during times of crisis.

### Day 3 – PPC at the macro-level

On the final day of the workshop, the focus broadened to tackle strategies for advancing PPC at the country and regional levels (macro-level). Through four plenary sessions and a collaborative group activity, participants explored key priorities for expanding interdisciplinary PPC through action plans to address regional needs.

The day began with welcoming five Morocco government officials and setting the stage for rich discussions around scaling PPC initiatives at the macro-level. The officials were: Dr Fadoua Rahhaoui, Dr Latifa Belakhel, Dr Loubna Abouselham and Dr Aasmaa Chaoui representing the Ministry of Health; and Professor Maria Bennani representing Lalla Salma Foundation. Their perspectives reinforced the importance of national strategies in advancing PPC, noting that palliative care for children in Morocco is manageable and can be a model for an effective approach in the region.

The insightful overview of PPC development in the region illustrated the inconsistent PPC growth across countries and the potential opportunities for collaboration to face common challenges. Particularly, workforce shortages, limited access to opioids and lack of specialised training and resources stimulated vibrant discussions on collective solutions. The next panel session further elaborated on engaging partners in the regional development of PPC. This panel was moderated Dr Michael McNeil and included Dr Giuseppe Troisi, Dr Dolly Noun, Ms Suheir Rasul, Dr Anwar Al-Nassan, Prof. Julia Downing and Dr Laila Hessissen. Their insights brought perspectives from St. Jude Global, governments, charitable foundations, POEM institutions, the WHO regional office and ICPCN. The panellists emphasised the importance of aligning with international standards, leveraging professional training opportunities, empowering regional champions from various disciplines, engaging local communities including religious figures, sharing success stories to reflect on cultural aspects and exploring collaborative research opportunities. Prof. Julia Downing then shared her personal journey in PPC leadership and advocacy, as a real-life example that an impactful change would require stepping out of our comfort zone and embracing uncertainty.

Building upon the need to extend partnerships, the ALSAC global team highlighted the critical role foundations have in strengthening PPC services. Participants shared their own experiences in working with foundations to support PPC. Stories included the ambassador programmes from Indus Hospital and Health Network in Pakistan and the Association of Friends of the National Cancer-free Initiatives in Egypt. Conversations highlighted the power of social media in raising awareness about childhood cancer and palliative care.

The afternoon culminated in a group activity where participants collaboratively prioritised barriers that are important and feasible to overcome in the region. The list of barriers was obtained from the study on Assessing Doctors Attitudes on Palliative Treatment that has been conducted in global settings [43, 44]. Participants prioritised respectively: professionals’ desire to maintain hope, cultural differences between patients or families and healthcare professionals, lack of trained professionals and family resistance. Compared to previous reports [43, 44], cultural differences distinguished the set of prioritised barriers, suggesting a specific cultural context for PPC worth exploring in the Eastern Mediterranean region. To address the prioritised barriers, participants identified current strengths and opportunities for growth within their healthcare systems at the micro-, meso- and macro-levels. Table 2 summarises the prioritised barriers along with the regional strengths and initial plans to address them at the micro-, meso- and macro-level. Participants developed a list of PPC activities to carry at the micro-, meso- and macro-levels combining various timelines. Examples of the discussed actions included: creating institutional policies and clinical guidelines; sensitising to PPC through community-level education campaigns and patient/family education materials; leveraging social media; developing PPC training programs at all levels; creating mentorship networks with local, regional and international leaders; and promoting PPC legislations.

The closing reflections on the workshop underscored the achievements made in 3 days and the collective commitment to advancing PPC at every level. Three main points summarise the macro-level aspects:

- Developing national strategies for PPC requires mobilising advocates from various sectors, including foundations and communities.
- The national and regional collaborations sparked during the workshop enhance continued dialogue and the sharing of successful solutions.
- Establishing national, regional and international partnerships can accelerate progress in addressing prioritised PPC barriers.

## Participants’ perspectives

**Figure 2.**
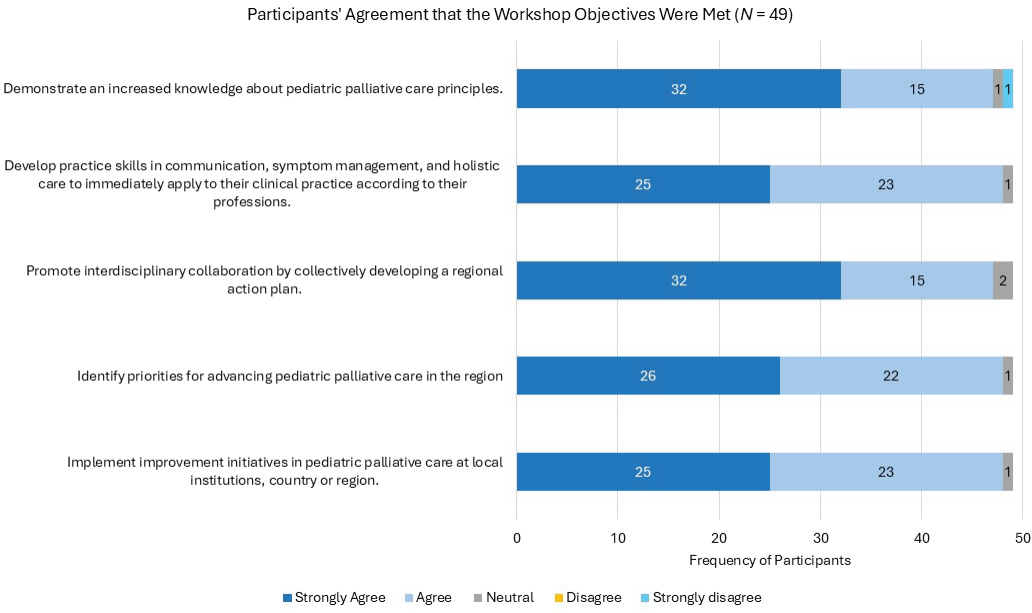
Participants’ agreement on meeting the workshop objectives. Figure 2 illustrates the frequency of participants’ responses on each level of agreement with meeting the objectives of the workshop. Out of the 49 participants who filled the evaluation survey, 47 ‘agree’ or ‘strongly agree’ that all the workshop objectives were met.

A total of 49 out of 52 regional participants completed the workshop evaluation (response rate: 94%). Most respondents (*n* = 42, 86%) had more than 2 years of experience in the care of children with cancer. However, only 16 (32%) from various countries in the region had received formal PPC training prior to the workshop. Most of the previously trained participants were physicians (*n* = 10), suggesting that training opportunities may be available in the countries, yet they may be more accessible to physicians than to other healthcare professionals. The overwhelming majority ‘strongly agreed’ or ‘agreed’ that the workshop objectives were met (Figure 2), and 98% (*n* = 49) rated the overall content as ‘Excellent’ or ‘very good’. Almost three-quarters (*n* = 38, 73%) reported that more than half of the workshop content was new to them. This high proportion highlights the need for training across disciplines and countries. In addition to the insufficient development of PPC in the region [9], this lack of training may also be attributed to regional instabilities and economic constraints that shift priorities of clinicians from professional development to survival.

More than 88% (*n* = 43) of participants ‘agreed’ or ‘strongly agreed’ that all the workshop sessions enhanced their knowledge. The session on ‘Importance of spiritual care’ had the lowest percentage (88%) highlighting a need for future educational activities among all disciplines and countries in the region. Over 91% expressed agreement on increased confidence in practicing specific PPC skills after participating in the workshop, with the lowest percentage for ‘managing physical symptoms other than pain’. Taken together, these findings suggest additional training needs, particularly in addressing cultural features of the region given the diversity in spiritual practices and in the availability of resources for symptom management.

In the free text responses, regardless of their professional background or country, participants recommended additional topics to enhance the workshop content. The suggestions included adding more content on communication skills, elaborating on the roles of the psychologist and social worker, including research conduct, addressing integrative medicine, and inviting patients and parents to share their experiences.

When asked about their projected steps after the workshop, participants shared their plans to initiate PPC teams or units, apply pain management, build communication skills, adopt a multidisciplinary approach, and involve parents in decision-making processes. They also expressed intentions to train colleagues in the core principles of PPC, conduct research on the perceptions about PPC and mapping regional resources along with maintaining collaborations through periodic networking meetings. This feedback aligns with the concrete plans of PPC growth opportunities identified during the last day of the workshop (Table 2).

The overwhelmingly positive feedback reflected an increase in PPC knowledge and confidence as reported by participants. Importantly, the workshop experience also sparked a shared appreciation of diversity, rooted in unifying goals and human experiences in PPC. One participant summarised the sentiment: ‘*I love to thank you all for this workshop that shows that PPC is a right to each patient and despite our differences in countries and cultures we all share the same goal and feeling*’.

## Implications

The workshop marked a pivotal milestone in advancing PPC in the Eastern Mediterranean region, highlighting the urgent need for PPC integration for children needing palliative care, including those with cancer. While surfacing regional disparities in PPC access, this event underscored the possible collaborations to leverage existing resources and the exchange of successful experiences in practice, education, research, policy and advocacy. By concerting efforts from key regional stakeholders notably POEM group, WHO-EMRO and regional referral centres, with the global support from St. Jude Global and ICPCN, PPC progress can be made. As Ms Suheir Rasul aptly stated, ‘*You are not alone. Together we can make the impossible possible*’.

At the patient-care level, this workshop equipped healthcare providers across disciplines with the skills to offer pain relief, symptom management, psychosocial and spiritual support and individualised communication throughout the disease journey. The immediate application of this knowledge and tools is encouraged to enhance patient care. As Prof. Julia Downing emphasised, ‘*If we do good palliative care, the patients and families become the advocates and spread the word on how PPC enhances quality of life, every little thing we do can induce a huge effect*’. While the practice of PPC can be challenging, the workshop fostered a sense of community among professionals who share similar passions, realities and hopeful outlooks. Such momentum planted the seed for creating a network of professionals dedicated to advancing PPC in the region.

At the educational level, PPC training remains a pillar for paradigm shift among healthcare providers and for raising awareness among patients, families and communities. Embedding PPC education into in-service training and university curricula cultivates a new generation of providers who are prepared to address the unique PPC needs of children, including those with cancer. Emerging regional initiatives such as the PPC fellowship in Jordan and academic interprofessional postgraduate programme in Lebanon promote specialist-level education and prepare regional mentors in the field. Providing simple education about PPC to patients and families can promote their awareness, understanding and demand of the care as their ‘human right’ [3].

In response to the global gap in PPC research from LMICs, this workshop unfolded the various attempts to understand the PPC landscape in the region. It marks a timely moment to amplify PPC mapping in the region and consolidate fragmented data to develop comprehensive reports of PPC activities, resources, barriers and successful solutions. Such collective understanding enables proper channelling of actions and engagement of regional stakeholders through the WHO regional office, the POEM group [19], the St. Jude Global Alliance [18] and ICPCN [22].

At the policy level, governments are urged to establish national frameworks that support PPC as a right for all children, ensuring equitable access. The PPC national strategy in Morocco is an exemplar of how governments along with professionals and foundations can advocate, support and provide guidance [45]. More regional advocacy efforts can mobilise policymakers and encourage collaborations across borders to share resources, expertise and best practices. Very recently, Gafer *et al* [46] emphasised the attributes of a successful model for integrating palliative care into Eastern Mediterranean using a primary care approach. Strengthening national, regional and international partnerships among healthcare professionals, charitable foundations and governments will lay the foundation for sustainable integration of PPC, ultimately improving the quality of life for children and their families across the region.

By conducting this workshop, we sought to generate momentum to address the pressing need to alleviate the suffering in the region associated with serious illness in children. To sustain the impact created by the workshop, we propose developing a roadmap of activities and projects led by regional experts with the support of WHO regional office and international collaborators. This roadmap will serve to track intentional efforts in regional PPC initiatives in education, clinical care, research and advocacy. Burgeoning efforts in education and research are underway, and this intentional engagement provides a space for learning and engagement not just within an institution or country, but throughout the region. Additionally, the workshop triggered valuable dialogue between the WHO, St. Jude Global, ICPCN, regional foundations and institutions to influence national and institutional policies for improving access to PPC. To maintain progress in these initiatives, we recommend reconvening in regional conferences including the bi-annual POEM scientific meetings, and in periodic PPC workshops to follow and refine the strategies. Finally, the workshop established a network of attendees to maintain an active dialogue, exchange expertise and celebrate achievements. The use of this network for regular updates of ongoing local, regional and international PPC initiatives can foster ongoing collaboration.

Moving forward, the plans for PPC development sparked during the workshop will be carried out by participants. Many challenges may arise in initiating PPC programs, training and introducing clinical guidelines. A common obstacle to early PPC integration is the confusion between palliative care and end-of-life care, which affects clinicians, communities and patients/parents [47]. Healthcare professionals driving PPC changes may encounter limited institutional support and workforce shortages due to shifting leadership priorities. In the regional context, systemic barriers such as opioid availability, regulatory restrictions and inadequate funding further constrain progress. These factors can hinder the ability to provide high-quality services. However, by building on the workshop experience and leveraging connections with regional experts, these challenges can be addressed. Coordinated efforts and the exchange of successful strategies in advocating for and implementing PPC reforms enable continuous progress.

## Conclusion

Over 3 days, the First Regional Interdisciplinary PPC Workshop in Rabat, Morocco engaged regional PPC providers, experts, foundation representatives and stakeholders in impactful discussions on expanding quality PPC in the Eastern Mediterranean region. Through collaborative learning, design thinking and knowledge sharing, the workshop served as a timely initial step toward collective planning to advance and sustain PPC integration for children with cancer. This first-of-a-kind workshop supported the journey of regional PPC change agents at the patient care, institutional and national/regional levels. This event highlighted opportunities to build partnerships and to transform the dire need for PPC into concrete actions that can improve the quality of life for children with cancer and their families in the region.

## Conflicts of interest

The authors declare that there are no conflicts of interest.

## Funding

This workshop is partially funded by American Lebanese Syrian Associated Charities (ALSAC) and the WHO Global Initiative for Childhood Cancer (GICC). There was no funding for the preparation of this report.

## Author contributions

RSR and RRL wrote the manuscript. All authors engaged in the design of the report and reviewed and approved the manuscript.

## Figures and Tables

**Table 1.** Demographic characteristics of participants (*n* = 52).

Characteristic	Number	Proportion (%)
Disciplines		
Physician ^a^	23	44
Nurse ^a^	19	37
Psychologist ^a^	4	8
Charitable foundation representative	3	6
Pharmacist	2	4
Social worker	1	2
Years of experience in the role (*n* = 49)		
< or = 1	7	14
2–5	17	35
6–10	10	20
11–15	8	18
> 15	7	12
Countries		
Morocco	19	37
Pakistan	8	15
UAE	4	8
Egypt	4	8
Palestine	4	8
Oman	3	6
Jordan	2	4
Kuwait	2	4
Lebanon	2	4
Türkiye	2	4
Qatar	1	2
Tunisia	1	2

Table 1 presents the frequency and proportion of each characteristic among participants with regards to distribution of disciplines, years of experience and countries represented

a One participant is a Charitable Foundation Representative in addition to his/her healthcare profession

**Table 2.** Strengths and future plans for PPC development in Eastern Mediterranean Region. (Continued)

Prioritized barriers	Micro-level		Meso-level		Macro-level	
	Strengths	Plans	Strengths	Plans	Strengths	Plans
Professionals’ desire to maintain hope	Desire to help others Religion The Will Passion for the profession Humanised care	Desire to help others Education of professionals Family involvement Exploring family desire Support group	Desire to help others Adult Palliative Care Mobile clinic for adult	Desire to help others Hiring social workers Ensuring availability of medication Patient/family satisfaction survey Interdisciplinary approach	Desire to help others Guide for palliative care GICC	Desire to help others Telemedine and Teleconsultation Exchange of experience Evaluate results Conduct national workshops Awareness campaigns on PPC benefits
Cultural differences between patient/families and professionals	Desire to help others Respect of family beliefs Empathy and compassion Open Mindedness	Desire to help others Patient advocacy Communication skills training Use of Artificial Intelligence in translation	Desire to help others Spiritual support Social worker support Psychosocial support No refusal policy Options for location of care	Desire to help others Collaboration and networking between institutions Public relation officers Diverse hiring practices Local language resources Cultural Assessment tools	Desire to help others Crescent of care model training	Desire to help others National policies and guidelines Cultural competency training
Lack of trained professionals	Desire to help others Communication skills Pathways for PPC exist in institutions Specialized Skills in HCP in some institutions Networking within the community Compassionate staff	Desire to help others Case discussions (challenging cases) Multidisciplinary rounds weekly/monthly Journal clubs Advocacy training for any healthcare professional Mentorship Specialized PPC education	Desire to help others Training programs for the physicians and nurses Training of nurses and nurse aids Hospital-level certificate courses Healthcare students’ rotation in PPC	Desire to help others Online training in different languages Continuing education post-graduation Training for healthcare professionals at the undergraduate level Local workshops Awareness sessions Collaboration between institutions	Desire to help others Some formal training on PPC Rotation of residents International PPC Fellowship Residents training and rotation in neighboring countries Accreditation (JCI)	Desire to help others National recognition of PPC training PPC certification
Family resistance	Families interested in cure and hope Wishes for patient Spiritual care	Support groups Patient & family education (consider language differences)	Child life specialist Psychosocial support	Training clinicians and caregivers Spiritual support International Fellowships Hospital beds policies Interdisciplinary team Shared decision-making Integrating PPC early at diagnosis Communication skills training, Practice truthtelling	Educational platforms and social media Policies in some communities Collaboration with charitable foundations	National guidelines, policies and regulations to facilitate access to medications Financial support Collaboration between organizations Use of social media for awareness campaigns Fundraising Financial and social support for patients/families

Table 2 summarises the prioritised barriers to Pediatric Palliative Care in the Eastern Mediterranean Region, the existing strengths and the initial plans to mitigate each barrier at the micro-, meso- and macro-level
